# Bacilloscopy and polymerase chain reaction of slit-skin smears and anti-phenolic glycolipid-I serology for Hansen’s disease diagnosis

**DOI:** 10.3389/fmed.2022.972244

**Published:** 2022-08-10

**Authors:** Filipe Rocha Lima, Natália Aparecida de Paula, Mateus Mendonça Ramos Simões, Gabriel Martins da Costa Manso, Gustavo Sartori Albertino, Giovani Cesar Felisbino, Vanderson Mayron Granemann Antunes, Fernanda André Martins Cruz Perecin, Andrezza Telles Westin, Helena Barbosa Lugão, Marco Andrey Cipriani Frade

**Affiliations:** ^1^Healing and Hansen’s Disease Laboratory, Ribeirão Preto Medical School, University of São Paulo, São Paulo, Brazil; ^2^Dermatology Division, Department of Internal Medicine, National Referral Center for Sanitary Dermatology and Hansen’s Disease, Clinical Hospital of the Ribeirão Preto Medical School, University of São Paulo, São Paulo, Brazil

**Keywords:** Hansen’s disease, bacilloscopy, slit-skin smear, PCR, serology, diagnosis

## Abstract

The bacilloscopy of the slit-skin smear (SSS) is the exclusive laboratory test associated with dermato-neurological evaluation for Hansen’s disease (HD) diagnosis; however, it is negative in the majority of PB or primary neural forms. Thus, a PCR technique involving different sequences and target genes has been performed with an aim to increase the sensitivity and specificity of *M. leprae* identification, especially in patients with low bacillary loads. Additionally, serological assays based on antibody response reflect infection levels and indicate that this could be a simpler, less invasive technique for estimating *M. leprae* exposure. Serological tests and PCR have been shown to be more sensitive and accurate than the SSS. Our study aimed to measure accuracy and performance among the SSS and PCR of dermal scrapings stored on filter paper and APGL-I serology for diagnosis in HD. A cross-sectional study analyzing the medical records (*n* = 345) of an HD outpatient-dermatology clinic from 2014 to 2021 was conducted. Accuracy performance parameters, correlation, and concordance were used to assess the value among the SSS, PCR, and APGL-I exams in HD. The SSS presented 24.5% sensitivity, 100% specificity, 37.4% accuracy, and the lowest negative predictive value (21.5%). The PCR assay had 41, 100, and 51% sensitivity, specificity, and accuracy, respectively. PCR and APGL-I serology increased the detection of HD cases by 16 and 20.6%, respectively. PCR was positive in 51.3% of patients when the SSS was negative. The SSS obtained moderate concordance with PCR [*k*-value: 0.43 (CI: 0.33–0.55)] and APGL-I [*k*-value: 0.41 (CI: 0.31–0.53)]. A moderate positive correlation was found between the APGL-I index and the bacillary index (*r* = 0.53; *P* < 0.0001). Thus, the use of the SSS is a low sensitivity and accuracy method due to its low performance in HD detection. The use of PCR and serological tests allows for a more sensitive and accurate diagnosis of patients.

## Introduction

HD is a treatable infectious disease with a chronic evolution, and its etiologic agent is the slow-growing organism *Mycobacterium leprae* complex, which includes *M. leprae* and *M. lepromatosis*. The bacteria compromise mainly the skin and peripheral nerves and can leave serious sequelae when there is no diagnosis or early therapeutic intervention (1). There were more than 127,000 new cases detected globally in 2020, a reduction in new case detection by 37% in 2020 compared with 2019, as an important consequence of the COVID-19 pandemic for HD control programs (2). According to WHO guidelines (2018), in addition to the cardinal clinical signs for the diagnosis of HD, the only remaining microbiological diagnosis test of HD is based on the presence of acid-fast bacilli in a slit-skin smear (SSS) even though this test is associated with low diagnostic accuracy for paucibacillary (PB) HD. Although the guidelines also reported polymerase chain reaction (PCR)-based assays as being associated with higher diagnostic accuracy, they lack standardization, are not commercially available, and would be difficult to perform in most primary health-care settings (3). Thus, the need to intensify the development and improvement of current laboratory methodologies for the early and satisfactory diagnosis of the disease is evident (4, 5). However, different clinical, bacteriological, and immunopathological characteristics constitute the spectrum of the disease and express the relationship between the pathogenicity of the bacilli and the host’s immune response, which makes it difficult and unfeasible to control the disease (6). Additionally and focusing on strategies to break the chains of HD transmission, WHO included a recommendation to implement HD post-exposure prophylaxis with single-dose rifampicin (SDR) for healthy close contacts of patients with HD aged 2 years or older, after excluding HD and tuberculosis diagnosis. However, the introduction of SDR has not yet been widely implemented and still being evaluated the benefits, costs, and risks of such interventions (7).

The SSS is practically the exclusive etiological diagnostic test for HD, and it is a low sensitivity method with numerous risks of misunderstandings and requires technical expertise in collection, fixation, staining, and reading (3). The SSS consists of a microbiological examination through Ziehl-Neelsen staining in smears of dermal scrapings from the earlobes, elbows, and knees as well as skin lesions to detect *M. leprae*, and its result is dependent on the patient’s bacillary load (8). The exam allows for classifying patients as multibacillary (MB) and monitoring the treatment when they are positive in the test (9). In general, the SSS is negative in the initial forms of the disease, pure neural cases, and some borderline cases, and it is strongly positive in the borderline lepromatous and lepromatous clinical forms (8). A negative SSS does not rule out the diagnosis of HD, and its sensitivity varies between 10 and 50% while its specificity is 100% (10). The performance of the test is based on the quality of the collection, the expertise of the performing professional and the laboratory protocol used to identify the entire bacteria (11).

The limitations in the diagnosis of PB and household contacts of HD patients (HDP) generate the need to incorporate techniques with greater technological performance with the aim of identifying groups of difficult bacilloscopic and histopathological diagnosis as a way to obtain an early diagnosis with high specificity (12). Currently, different gene targets are studied to support the specific detection of *M. leprae*. The gene sequence of specific regions located along the mycobacteria genome has become a potential and promising target for the molecular diagnosis of HD. Therefore, conventional or quantitative PCR assays provide fast and reliable results for molecular detection and/or quantification (13). PCR is sensitive and 100% specific due to negative results in samples of different mycobacteria, healthy individuals, and other granulomatous diseases (14). Thus, PCR can be used as a complementary test for the diagnosis of HD regardless of the clinical form of the disease (13, 14). The type of material collected, means of transport, technique chosen, and targets for detection of the bacteria are the main variables influencing the results (15). PCR of dermal scrapings stored in 70% alcohol or from skin biopsies has been highlighted, but its transport poses risks. Therefore, new ways of collecting and storing the collected sample are being validated, such as PCR of dermal scrapings on filter paper, as we have used at our hospital over the last 10 years.

Considering that for both the SSS and PCR the dermal scrapings come from the same closed place, without contact with the environment and other contaminants, such as from nasal and/or oral mucosa, the search for specific *M*. *leprae* DNA (PCR) tends to offer better positivity than the search for morphologically complete bacilli (SSS), even in low-load early cases (16).

Additionally, by serology, high titers of IgM anti-phenolic glycolipid-I antibodies (APGL-I) are associated with dissemination and progressive infections by the bacilli, making the test positive preferentially in MB cases of high burden, but its detection for PB patients is of limited value (4). Although very widespread in the literature, serological studies with PGL-I antigen have shown an average sensitivity of 63.8 and an average specificity of 91% (17). Thus, our study aimed to measure accuracy and performance among the SSS and PCR exams of dermal scrapings collected on filter paper and APGL-I serology in the diagnosis of HD.

## Materials and methods

### Design and study population

A cross-sectional study was conducted at the National Reference Center in Sanitary Dermatology and HD, Clinical Hospital of Ribeirão Preto Medical School (HCFMRP-USP), University of São Paulo, Brazil. Medical records (*n* = 345) of the HD outpatient-dermatology division from 2014 to 2021 were reviewed. The inclusion criterion for the selected patients was the execution of the three exams evaluated (SSS, PCR, and APGL-I) at the same time. The medical records were classified according to diagnosis into two patient groups by clinical screening of HD, as new cases of HD without multidrug therapy (MDT) and patients without diagnosis of HD who presented some skin lesions or neuropathy. Both groups underwent the three tests compared in the study (SSS, PCR, and APGL-I) and clinical evaluation at the same time.

#### Hansen’s disease patients

HDP (*n* = 286) were diagnosed by clinical evaluation according to the WHO guidelines and recommended cardinal signs (3). The dermatological and neurological evaluation of the patients was the confirmatory exam for diagnosis of HD. Complementary tests to the clinical diagnosis were used when available as serology, molecular exam, bacilloscopy, ultrasound of peripheral nerves, electroneuromyography, and assessment of tactile sensation by Semmes-Weinstein esthesiometer. Negative results for complementary exams did not rule out the clinical diagnosis of HD. Clinical evaluations were performed by dermatologists and leprologists. Considering that none of the classifications for HD include all of the clinical manifestations of HD, particularly those involving macular and pure neural forms, we classified the patients considering the guidelines adapted by Madrid (Congress of Madrid 1953) and the Indian Association of Leprology (IAL 1982) classifications as follows: indeterminate (I), polar tuberculoid (TT), borderline (B), polar lepromatous (LL), and pure neural (N).

#### Non-Hansen’s disease patients

Non-Hansen’s disease patients (N-HDP) (*n* = 59) were defined as patients presenting some skin lesions or neuropathy who were referred due to the suspicion of HD. The signs and symptoms of HD suspicion were dermatological such as skin lesions with altered thermal, painful and/or tactile sensation, nodules, loss of eyelashes and/or eyebrows, as also, neurological findings such feel numbness in hands or feet, tingling (pricking), stinging sensation, pain in the nerves, weakness in the hands and/or feet, swelling of hands, feet and/or face. After dermato-neurological evaluation and complementary laboratory tests, these patients had the diagnosis of HD excluded and were classified as N-HDP.

### Bacilloscopy

The SSS remains the reference standard of HD detection and is taken from 4 routine sites of dermal scraping samples from earlobes and at least one elbow and/or typical skin lesion. According to the Brazilian Ministry of Health guidelines, bacterial index (BI) counting and morphological analysis were used in a common optical microscope. The Ziehl–Neelsen technique was applied on the intradermal scraping slide containing four smears from each patient. The BI is an index of the bacillary load in the patient. This is expressed on a semilogarithmic scale: (1 +) 1–10 bacilli per 100 high-power (oil immersion) fields, (2 +) 1–10 bacilli per 10 high-power fields, (3 +) 1–10 bacilli per high-power field, (4 +) 10–100 bacilli per high-power field, (5 +) 100–1,000 bacilli per high-power field and (6 +) > 1,000 bacilli per high-power field (8).

### Anti-phenolic glycolipid-I serology

Indirect ELISA was used as index test to measure the APGL-I IgM titer of every serum sample according to a previously reported protocol (4). Serology was performed with ND-O-BSA (PGL-I) based glycoconjugate of bovine serum albumin (NR-19346. BEI Resources). The respective index was calculated by dividing the optical density (O.D. 450 nm) of each sample by the cutoff, and indices above 1.0 were considered positive.

### Polymerase chain reaction for detecting *Mycobacterium leprae* DNA

PCR was used as second index test. Dermal scraping samples from earlobes and at least one elbow and/or lesion obtained in the same collection performed for the SSS were transferred and stored on filter paper. Samples were refrigerated at 2–8°C until processing. Filter paper with samples was cut with a scalpel blade and transferred to a microtube, and 100 μL of sterile Milli-Q water was added. For material elution, the tube was incubated at 95°C for 15 min. Total DNA extraction was performed with commercial DNA extraction according to the manufacturer’s protocol. DNA was used to perform conventional or quantitative PCR with primers specific to *M. leprae* according to a previous study (4). For conventional PCR the band was used to identify the PCR product with molecular weight relative to positive control with 148 bp. The quantitative PCR (qPCR) result was considered positive to detect *M. leprae* DNA with amplification until 40.0 cycle threshold (Ct) and melting temperature at 87.5°C.

### Statistical analysis

All data were analyzed by GraphPad Prism v. 9.0 software (GraphPad Inc., La Jolla, CA, United States). The chi-squared test was used to assess associations among categorical variables and the positivity of exams. Study population characteristics were analyzed by a *t*-test and chi-squared test. Spearman’s correlation was used to compare the immunoglobulin index APGL-I and BI in the SSS. The kappa coefficient (κ) was used to measure the reliability and concordance of the SSS, molecular test (PCR), and serology (APGL-I). The interpretation used for kappa value was slight (0-0.2), fair (0.21-0.4), moderate (0.41-0.6), substantial (0.61-0.8) and almost perfect (0.81-1.0). The level of statistical significance was set by alpha value (5%). Venn diagrams were generated using the online tool Draw Venn Diagram^1^ to represent the overlap in the number of positive exams in the HDP. According to Bossuyt et al. (9), the Standards for Reporting of Diagnostic Accuracy Studies (STARD statement) was used to improve the completeness and transparency of results of diagnostic accuracy available at: https://www.equator-network.org/reporting-guidelines/stard/ (9). According to Whiting et al. (10), risk of bias and applicability judgments was applied using Quality Assessment of Diagnostic Accuracy Studies (QUADAS-2) available at: https://www.bristol.ac.uk/population-health-sciences/projects/quadas/quadas-2/ (10). The study was developed with pre-specified tests and considering SSS as reference standard and, PCR and APGL-I as index tests.

## Role of the funding source

The funder of the study had no role in the study design, data collection, data analysis, data interpretation, or writing of the report. All authors had full access to all of the data in the study and had final responsibility for the decision to submit for publication.

## Results

### Assessment of completeness, transparency, and risk of bias

**FIGURE 1:**
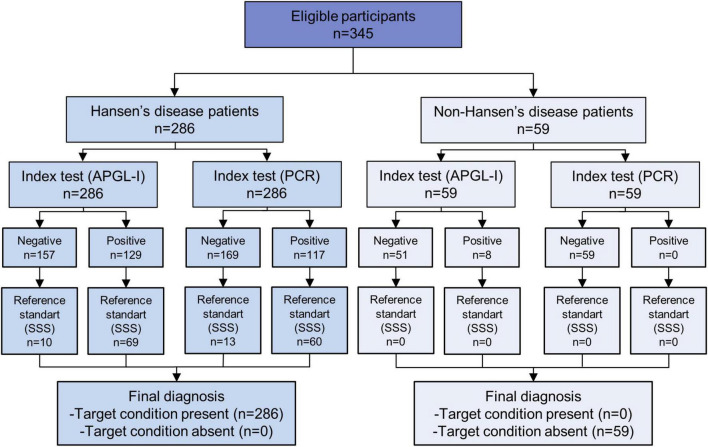
STARD diagram to report flow of participants through the study.

To determine the quality and standardize the developed accuracy study, the STARD and QUADAS-2 were applied. STARD diagram to report flow of participants through the study was designed to summarize the selection of the population and performance of the index tests (APGL-I and PCR) as compared to the standard reference test (SSS) (Figure 1). All participants performed the standard reference test and the two index tests. In our study, inconclusive results were not reported. The STARD checklist was completed to assess the transparency of the study (Supplementary Table 1). The analysis of risk of bias and regarding applicability showed a concern classified as low for bias (Supplementary File).

### Clinical and demographic characteristics

The study included 345 medical records grouped as HDP (*n* = 286; 82.9%) and N-HDP (*n* = 59; 17.1%). No significant difference was observed among ages (*P* = 0.363) and between genders (*P* = 0.645) of the groups. Among the 286 HDP, 197 (68.9%) were classified as B clinical forms of HD, 16 (5.6%) were classified as I and N forms, 9 (3.1%) as TT form, and 48 (16.8%) as LL form. The descriptive characteristics of the study population are summarized in Table 1.

**TABLE 1:** Study population characteristics (*N* = 345).

	HDP *n* = 286	N-HDP *n* = 59	*P*
Age, years, mean (SD)	49.4 (16.1)	49.8 (15.9)	0.363^a^
**Sex, *n* (%)**			
Male	180 (62.9)	39 (66.1)	0.645^b^
Female	106 (37.1)	20 (33.9)	
**Clinical form**	***n* (%)**	***n* (%)**	
Indeterminate	16 (5.6)	-	
Tuberculoid	9 (3.1)	-	
Borderline	197 (68.9)	-	
Lepromatous	48 (16.8)	-	
Neural Pure	16 (5.6)	-	

^a^Comparison of two groups using the t-test. ^b^Comparison of two groups using the chi-squared test. HDP, HD patients; N-HDP, non-HD patients; SD, standard deviation.

### Performance of laboratory tests in diagnosis of Hansen’s disease

An analysis of the performance parameters of laboratory tests was performed to assess the concordance for the SSS, PCR on filter paper, and APGL-I serology for the diagnosis of HD patients (Table 2). The SSS presented a probability of case detection of only 24.5 and 100% specificity. Thus, it presented the lowest negative predictive value (21.5%), accuracy (37.4%), and positivity for evaluated cases (24.5%). The PCR assay had the best significant performance (*P* < 0.0001), with 41, 100, and 51% sensitivity, specificity, and accuracy, respectively. APGL-I had the highest probability of detecting cases (41%) and providing correct results (52.2%) and the highest rate of positive tests in patients (45.1%) despite yielding a positive test in individuals without diagnosis of HD (13.6%). The use of dermal scrapings for PCR and APGL-I serology increased the detection of HD cases to 16 and 20.6%, respectively. The SSS and PCR are the only tests that showed 100% probability of the disease when the test was positive. Positivity for all exams separately and in parallel was significantly different compared with the N-HDP (*P* < 0.0001).

**TABLE 2:** Comparison of the performance for the SSS, PCR and APGL-I as diagnostic tests in HD.

						Positivity		
						
Exams	Se%	Sp%	PPV%	NPV%	Acc%	HDP *n* (%)	N-HDP n (%)	*P* ^a^
SSS	24.5	100	100	21.5	37.4	70 (24.5)	0 (0)	**<0.0001**
PCR	41.0	100	100	25.9	51.0	117 (41)	0 (0)	**<0.0001**
APGL-I	45.1	86.4	94.2	23.8	52.2	129 (45.1)	8 (13.6)	**<0.0001**
SSS and PCR	26.8	100	100	27.4	42.6	57 (19.9)	0 (0)	**<0.0001**
SSS and APGL-I	29.0	100	100	28.6	44.7	60 (21.0)	0 (0)	**<0.0001**
PCR and APGL-I	39.6	100	100	33.7	53.8	76 (26.6)	0 (0)	**<0.0001**
SSS + PCR-	7.7	100	100	27.4	31.6	13 (4.5)	0 (0)	**0.028**
SSS- PCR +	27.8	100	100	27.4	43.3	60 (21.0)	0 (0)	**<0.0001**
SSS + APGL-I -	6.4	100	100	28.6	33.5	10 (3.5)	0 (0)	**0.047**
SSS- APGL-I +	50.0	86.4	89.6	25.9	43.8	69 (24.1)	8 (13.6)	**0.005**
PCR + APGL-I -	25.6	100	100	33.7	40.3	40 (14.0)	0 (0)	**<0.0001**
PCR- APGL-I +	31.4	86.4	86.9	30.5	45.6	53 (18.5)	8 (13.6)	**0.007**

^a^Chi-squared test between HD patients (HDP; n = 286) and non-HD patients (N-HDP; n = 59) positivity. For the serial evaluation, the + and - signs were used to represent positive and negative results, respectively. SSS, slit-skin smear; PCR, polymerase chain reaction; APGL-I, IgM anti-phenolic glycolipid-I; Se, sensitivity; Sp, specificity; PPV, positive predictive value; NPV, negative predictive value; Acc, accuracy.

The evaluation of tests in parallel showed that the inclusion of the SSS with PCR reduced by 20% the detection of cases and had 29 and 44.7% of sensitivity and accuracy, respectively. The absence of positive results for SSS and PCR in N-HDP maintained 100% specificity and positive prediction of the tests. PCR positivity and APGL-I serology combined demonstrated the best sensitivity (39.6%), negative predictive value (33.7%), accuracy (53.8%), and positivity (26.6%), when compared to performance including SSS (Table 2).

Serial analysis with positive SSS when the results were negative for PCR and APGL-I had the lowest sensitivity (7.7%; 6.4%) and accuracy (31.6%; 33.5), respectively. However, PCR performance in negative SSS results was 27.8% for probability of case detection and 43.3% of accuracy. Negative SSS results when the APGL-I serology was positive had the best sensitivity (50%); on the other hand, it had the lowest negative predictive value (25.9%). The results were similar for positive results for APGL-I serology when PCR was negative and reached the best accuracy (45.6%). The positivity in HDP for SSS when serology was negative had the lowest rate (3.5%; *P* = 0.047) (Table 2).

### Concordance among the slit-skin smear, polymerase chain reaction, and anti-phenolic glycolipid-I

Analysis of concordance among the three tests was applied to evaluate the positivity of these exams in HD cases (Table 3). The overlap of the SSS results with PCR and APGL-I achieved moderate concordance, with kappa values ranging from 0.43 (CI: 0.33–0.55) and 0.41 (CI: 0.31–0.53), respectively. The analysis between PCR and APGL-I serology showed a concordance classified as fair with a kappa value of 0.33 (CI: 0.22–0.44).

**TABLE 3:** Concordance among the SSS, PCR and APGL-I results in HDP.

Overlap	kappa (*k*) value	95% CI	Standard error	Interpretation
SSS vs. PCR	0.437	0.33–0.55	0.057	Moderate
SSS vs. APGL-I	0.418	0.31–0.53	0.056	Moderate
PCR vs. APGL-I	0.331	0.22–0.44	0.057	Fair

SSS, slit-skin smear; PCR, polymerase chain reaction; APGL-I, IgM anti-phenolic glycolipid-I; HDP, HD patients; CI, confidence interval.

### Positivity overlap for the slit-skin smear, polymerase chain reaction, and anti-phenolic glycolipid-I exams

**FIGURE 2:**
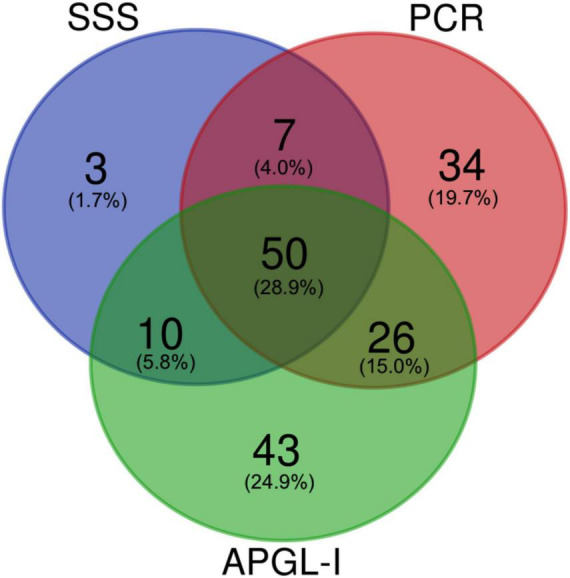
The Venn diagrams represent the overlap in the number of positive SSS, PCR, and APGL-I index examinations in HD patients. Data were represented in absolute value and percentage relative to the total of positive tests (*n* = 173/286). SSS, slit-skin smear; PCR, polymerase chain reaction; APGL-I, IgM anti-phenolic glycolipid-I.

The performance generated by the Venn diagram showed the positivity overlap of the SSS, PCR, and APGL-I tests (60.5%; 173/286 HDP), which was 28.9% (50/173) for all assays; 1.7% (3/173) were only positive for the SSS, and the highest number of positive results isolated were for PCR (19.7%; 34/173) and APGL-I (24.9%; 43/173). PCR and APL-I were superior methodologies for the diagnosis of HD as compared with the SSS exam, which showed less than 61.5 and 73.1% overlap with APGL-I and PCR, respectively, compared with APGL-I and PCR overlap (Figure 2).

### Correlation among anti-phenolic glycolipid-I serology, polymerase chain reaction, and bacillary load bacterial index from slit-skin smear

**FIGURE 3:**
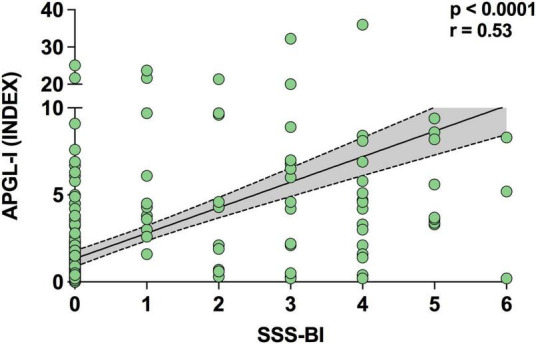
The APGL-I serology index shows a moderate positive correlation with the SSS bacillary load. Spearman’s correlation coefficient was used to compare the bacterial index in the SSS (BI-SSS) with the APGL-I index. SSS, slit-skin smear; APGL-I, IgM anti-phenolic glycolipid-I.

Correlation analyses were performed to assess the antibody levels and bacillary load. There was a moderate positive correlation between the index of APGL-I IgM and BI resulting from the SSS (*r* = 0.53; *P* < 0.0001) (Figure 3). The comparative evaluation between the positive PCR (PCR +) and negative PCR (PCR-) results in relation to BI-SSS showed that 92.3% (156) of HD PCR cases were also negative BI-SSS. PCR was positive in 51.3% (60) of the HDP when the BI-SSS was negative (BI = 0). The PCR results ranged from 1.2 to 2.4% in patients with BI-SSS scores between 1+ and 4+. HDP with BI-SSS 5 + and 6 + showed only PCR + results (Table 4).

**TABLE 4:** Comparison between PCR and bacillary load results in HDP.

BI-SSS	PCR– *n* (%)	PCR+ *n* (%)
0	156 (92.3)	60 (51.3)
1+	4 (2.4)	7 (6.0)
2+	4 (2.4)	9 (7.7)
3+	3 (1.7)	11 (9.4)
4+	2 (1.2)	19 (16.2)
5+	0 (0)	8 (6.8)
6+	0 (0)	3 (2.6)
Total	169 (100)	117 (100)

HD patients (HDP; n = 286). PCR, polymerase chain reaction; BI-SSS, bacterial index from slit-skin smear.

## Discussion

Our study was designed to evaluate the performance of three different laboratory assays (SSS, PCR, and APGL-I) to aid in the diagnosis of HD. The results obtained allowed the comparison between patients with HD (HDP) and patients referred to the outpatient clinic of dermatology with suspected HD due to dermato-neurological signs and symptoms, and the diagnosis of HD was ruled out through clinical and/or laboratory evaluation (N-HDP). The majority of HDP (68.9%) were classified as B clinical forms, and 85.7% were classified as MB forms (B and LL). Similarly, 67.3% of the cases reported worldwide by the WHO and 80.1% reported by the Brazilian Ministry of Health were MB diagnoses in 2020 and 2021, respectively (2, 18).

According to the WHO (3), the SSS is the only laboratory technique considered a cardinal sign used as a diagnostic criterion in bacteria identification, and it operationally classifies individuals between MDT PB and MB schemes when positive (3). In addition to being a tool that is highly dependent on the expertise of the performing professional and the patient’s bacillary load, the main difficulty for its routine use is the low sensitivity in the diagnosis of initial cases with low bacterial load and exclusively neural HD (16). Thus, the goals proposed by the WHO (2) of having early diagnosis and laboratory techniques that identify subclinical infection are unfeasible (19). The SSS showed sensitivity and positivity of only 24.5%, despite its high specificity (100%), and the technique is classified as having low accuracy (37.4%) for the diagnosis of HD. Siwakoti et al. (13) demonstrated that the SSS sensitivity can be 18%, and microscopy has the advantage of being easily available at peripheral and referral centers; however, as its detection limit is 10^4^ bacilli/ml, it suffers from low sensitivity (13). Additionally, Gurung et al. (20) reported that the SSS can have a sensitivity of 24–41% and a specificity of 93–100% (20).

The use of PCR and APGL-I techniques allowed an increase of 16.5 and 20.6%, respectively, for the identification of HD patients as compared with the SSS, although the SSS showed moderate concordance with the results of PCR and APGL-I serology. The quantitative evaluation of the SSS by BI shows a moderate positive correlation with APGL-I serology. On the other hand, 51.3% of the SSS results with negative BI had a positive PCR test. Thus, 38.5% of negative BI-SSS became positive in the PCR assay, a considerable improvement.

In the diagnosis of patients presenting only hypochromatic macular lesions investigations focusing almost exclusively on cutaneous signs, the inability of widely used tests to detect the different clinical forms and reaction states, and the identification of subclinical infection are challenges in controlling the magnitude of the disease and result in a non-timely diagnosis (14, 21, 22). The PCR technique consists of extracting, amplifying, and identifying *M. leprae* DNA in clinical samples, such as intradermal scrapings, with higher laboratory accuracy, and it mitigates the main gaps with the use of the SSS alone in laboratory diagnosis across the clinical spectrum of HD. A compilation of published studies reports PCR sensitivity ranging from 51 to 91% and specificity ranging from 46 to 100% (20). Currently, different gene targets are being studied to support the specific detection of *M. leprae*. The gene sequence of specific repetitive element regions (RLEP) located along the mycobacteria genome is an established target for the molecular diagnosis of HD. The application of molecular assays with RLEP allows greater sensitivity by providing multiple copies throughout the genome. Therefore, PCR-RLEP assays provide fast and reliable results for molecular detection and quantification (16, 23).

In addition to presenting a 16.5% greater ability to diagnose new cases compared to SSS, PCR showed 100% specificity and accuracy with a 36.5% increased chance of diagnosis when positive and compared with controls (N-HDP). Corroborating the findings of other published studies, Azevedo et al. (16) demonstrated that PCR was sensitive and 100% specific (16), while the detection of RLEP DNA in the SSS was higher when using qPCR, with 84% sensitivity, 75% specificity, and 77% accuracy (23). Thus, PCR-RLEP can be used as a complementary test for the diagnosis of HD regardless of the clinical form of the disease. The lower and variable sensitivity and accuracy of the molecular assay can be associated with the technique used (conventional or qPCR), gene target for identification of the bacteria, protocol used, and patients’ bacillary load. In our study, 93.9% of the results were obtained by conventional PCR. The evaluation of the test performance to identify bacteria DNA showed a difference of only 4% less positivity with the use of the qPCR technique.

Despite showing a fair concordance between PCR and serology results for the detection of APGL-I, we found a difference of only 4.1% in the ability to detect the disease between these tests. On the other hand, APGL-I serology was 13.6% positive in cases without a clinical diagnosis of HD. APGL-I positivity has been mainly correlated with MB forms and with higher BI-SSS scores (24). Thus, laboratory assays using APGL-I and anti-LID-1 by ELISA and rapid test platforms with NDO-LID show low values of specificity and sensitivity and are not recommended for isolated use in the diagnosis of HD, considering the complexity of immunological presentations, and disease clinics (25). The study published by Frade et al. (25) demonstrated a sensitivity ranging between 48 and 62% and a specificity of 70% for APGL-I and anti-LID ELISA and 40% for NDO-LID. Also, 30% positivity for APGL-I serology among individuals with no history of contact with an HD patient, indicative of the generally high rate of exposure in the endemic region (25). Worobec (26) showed that subclinical *M. leprae* infection in endemic regions with APGL-I seropositivity was detected in 1.7–35% of all individuals, in view that the PGL-I antigen used in serology is species-specific (26). In our study, N-HDP showed 13.6% APGL-I ELISA positive, considering that the city was classified in the last year (2021) as very high endemicity, even in a low endemic state since 2006 as São Paulo.

Most of the published studies use IgM as a target molecule in serological assays because the seroprevalence of anti-PGL-I IgM is higher than the seroprevalence of IgA and IgG in endemic areas (27). IgM seropositive individuals are at increased risk of developing the disease (28); however, IgM seropositivity is not predictive of disease, as demonstrated with IgG APGL-I (29, 30). Therefore, the APGL-I serological assay is limited because it cannot be used as a predictive criterion for the diagnosis of the disease when there is no association with the neurological and/or dermatological diagnosis of HD.

The use of the same collection sites (intradermal scraping) for PCR processing provides the same interpretation of the SSS as a microbiological diagnostic criterion, which is the identification of bacilli in the host. However, detecting *M. leprae* DNA using a modern technique with greater sensitivity and 100% specificity allows us to rethink the use of the SSS in outpatient routines, as it is a methodology that presents low positivity. It could not detect 75.5% of the HD cases evaluated in our study despite 85.7% of the patients being classified as having clinical forms B and LL considered operationally MB. Similarly, the low accuracy occurred even with cases with higher bacillary load or more advanced stages of the disease and who were diagnosed in tertiary-level centers and highly specialized research centers in a national reference service comprising dermatologists and leprologists.

As a way of assisting in the assessment of bacillary load and therapeutic monitoring of cases, the use of qPCR offers a quantitative method, making it possible to assess the amount of DNA in the sample through a calculation considering the *Ct*-value, volume of DNA extract, volume of template, and mean RLEP copy number (31). The presence of *M. leprae* DNA was best detected in skin biopsies and skin scrapings independent of the extraction method or the clinical form. Interestingly, skin scrapings are less invasive samples and are the second-best clinical sample type for *M. leprae* detection (32). A study published by Gobbo et al. (33) showed that increasing the number of Ct (>40) and using qPCR can provide greater sensitivity (86% positivity in HD cases), increasing the identification of early cases of the disease, household contacts and oligosymptomatic individuals (23, 33).

In view of the findings evidenced in the study, the use of the SSS as a way of diagnosing HD is presented as a low sensitivity and accuracy method due to its low performance in case detection. The use of molecular biology methodologies (PCR) and association with serological techniques allow for a more accurate clinical diagnosis of patients as well as identifying a greater number of individuals regardless of the clinical form or operational classification. Additionally, the need for implementation of serological techniques and PCR as complementary tests is not restricted to referral and research centers because we are delaying the diagnosis and treatment and consequently increasing the disability and stigma of HD patients. Therefore, bacilloscopy should be urgently rethought as the exclusive criterion for the laboratory diagnosis of HD as proposed by the WHO guidelines.

## Data availability statement

The raw data supporting the conclusions of this article will be made available by the authors, without undue reservation.

## Ethics statement

The studies involving human participants were reviewed and approved by the Institutional Review Board for Human Research of the HCFMRP-USP (MH-Brazil Project—Protocol number 16620/2014). The ethics committee waived the requirement of written informed consent for participation.

## Author contributions

FL and MF substantially contributed to manuscript conception and design, acquisition of data, and analysis and interpretation of data. HL, FP, and AW contributed to the clinical care of patients. FL, MS, GA, GF, and VA contributed to acquisition of clinical data and review of the medical records. FL and NP contributed to execution and interpretation of the laboratory tests. FL, GM, and MF contributed to the statistical analysis and interpretation of the data. MF gave final approval of the final submitted version and any revisions, as well as provided supervision and orientation of the study. All authors contributed to the interpretation of the results and critical revision.

## Footnotes

1
http://bioinformatics.psb.ugent.be/webtools/Venn/
