# Supplementary material for: Bacilloscopy and polymerase chain reaction of slit-skin smears and anti-phenolic glycolipid-I serology for Hansen’s disease diagnosis
Source: Front Med (Lausanne). 2022 Aug 10;9:972244. doi: 10.3389/fmed.2022.972244 (PMC9399463; doi:10.3389/fmed.2022.972244)
Supplement: Supplementary file 1 [file Data_Sheet_1.pdf]

Supplementary Table S1: STARD checklist

Section & Topic	No	Item	Reported on page #
**TITLE OR ABSTRACT**			
	**1**	Identification as a study of diagnostic accuracy using at least one measure of accuracy (such as sensitivity, specificity, predictive values, or AUC)	1
**ABSTRACT**			
	**2**	Structured summary of study design, methods, results, and conclusions (for specific guidance, see STARD for Abstracts)	1
**INTRODUCTION**			
	**3**	Scientific and clinical background, including the intended use and clinical role of the index test	2
	**4**	Study objectives and hypotheses	2
**METHODS**			
Study design	**5**	Whether data collection was planned before the index test and reference standard were performed (prospective study) or after (retrospective study)	3
Participants	**6**	Eligibility criteria	3
	**7**	On what basis potentially eligible participants were identified (such as symptoms, results from previous tests, inclusion in registry)	3
	**8**	Where and when potentially eligible participants were identified (setting, location and dates)	3
	**9**	Whether participants formed a consecutive, random or convenience series	3
Test methods	**10a**	Index test, in sufficient detail to allow replication	3
	**10b**	Reference standard, in sufficient detail to allow replication	3
	**11**	Rationale for choosing the reference standard (if alternatives exist)	3
	**12a**	Definition of and rationale for test positivity cut-offs or result categories of the index test, distinguishing pre-specified from exploratory	4
	**12b**	Definition of and rationale for test positivity cut-offs or result categories of the reference standard, distinguishing pre-specified from exploratory	4
	**13a**	Whether clinical information and reference standard results were available to the performers/readers of the index test	4
	**13b**	Whether clinical information and index test results were available to the assessors of the reference standard	4
Analysis	**14**	Methods for estimating or comparing measures of diagnostic accuracy	4
	**15**	How indeterminate index test or reference standard results were handled	Not applicable
	**16**	How missing data on the index test and reference standard were handled	Not applicable
	**17**	Any analyses of variability in diagnostic accuracy, distinguishing pre-specified from exploratory	4
	**18**	Intended sample size and how it was determined	4
**RESULTS**			
Participants	**19**	Flow of participants, using a diagram	5
	**20**	Baseline demographic and clinical characteristics of participants	5
	**21a**	Distribution of severity of disease in those with the target condition	Not applicable
	**21b**	Distribution of alternative diagnoses in those without the target condition	Not applicable
	**22**	Time interval and any clinical interventions between index test and reference standard	3
Test results	**23**	Cross tabulation of the index test results (or their distribution) by the results of the reference standard	6
	**24**	Estimates of diagnostic accuracy and their precision (such as 95% confidence intervals)	6
	**25**	Any adverse events from performing the index test or the reference standard	Not applicable
**DISCUSSION**			
	**26**	Study limitations, including sources of potential bias, statistical uncertainty, and generalisability	4
	**27**	Implications for practice, including the intended use and clinical role of the index test	6
**OTHER INFORMATION**			
	**28**	Registration number and name of registry	Not applicable
	**29**	Where the full study protocol can be accessed	Not applicable
	**30**	Sources of funding and other support; role of funders	9

QUADAS-2

Phase 1: State the review question:

Patients (setting, intended use of index test, presentation, prior testing):<br>New cases of Hansen's disease (HD)
Index test(s):<br>PCR to detect DNA and anti-PGL-I IgM (APGL-I) serology
Reference standard and target condition:<br>Bacilloscopy (Slit-skin smear - SSS). HD diagnosis

Phase 2: Draw a flow diagram for the primary study

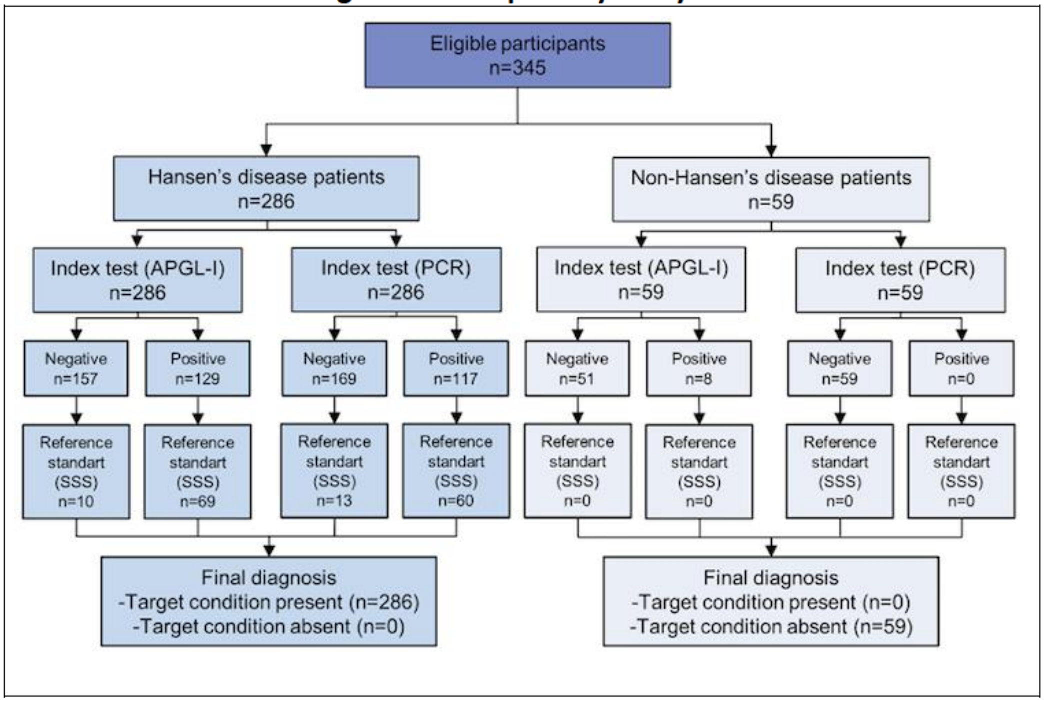

Phase 3: Risk of bias and applicability judgments

QUADAS-2 is structured so that 4 key domains are each rated in terms of the risk of bias and the concern regarding applicability to the research question (as defined above). Each key domain has a set of signalling questions to help reach the judgments regarding bias and applicability.

DOMAIN 1: PATIENT SELECTION

A. Risk of Bias

Describe methods of patient selection:

The inclusion criterion for the selected patients was the execution of the three exams evaluated (SSS, PCR and APGL-I) at the same time. The medical records were classified according to diagnosis into two patient groups by clinical screening of HD: new cases without multidrug therapy (MDT) and patients without HD diagnosis who presented some skin lesions or neuropathy.

- ❖ Was a consecutive or random sample of patients enrolled? Yes — Yes/No/Unclear
- ❖ Was a case-control design avoided? Yes — Yes/No/Unclear
- ❖ Did the study avoid inappropriate exclusions? Yes — Yes/No/Unclear

Could the selection of patients have introduced bias? **RISK: LOW/HIGH/UNCLEAR**

Low

B. Concerns regarding applicability

Describe included patients (prior testing, presentation, intended use of index test and setting):

New cases of HD and patients presenting some skin lesions or neuropathy who referred due to the suspicion of HD. After dermatoneurological evaluation and complementary laboratory tests, these patients had the diagnosis of HD excluded or confirmed. Clinical exam was the confirmatory test for the HD diagnosis.
Bacilloscopy (SSS) was the reference standard and, PCR and APGL-I were classified as index tests.

Is there concern that the included patients do not match the review question? **CONCERN: LOW/HIGH/UNCLEAR**

Low

DOMAIN 2: INDEX TEST(S)

If more than one index test was used, please complete for each test.

A. Risk of Bias

Describe the index test and how it was conducted and interpreted:

1. APGL-I: Indirect ELISA was used as index test to measure the APGL-I IgM titer of every serum sample.
The respective index result was calculated by dividing the optical density (O. 450 nm) of each sample by the cutoff, and indices results above 1.0 were considered positive.

- ❖ Were the index test results interpreted without knowledge of the results of the reference standard? Yes — Yes/No/Unclear
- ❖ If a threshold was used, was it pre-specified? Yes — Yes/No/Unclear

Could the conduct or interpretation of the index test have introduced bias? **RISK: LOW /HIGH/UNCLEAR**

Low

B. Concerns regarding applicability

Is there concern that the index test, its conduct, or interpretation differ from the review question? **CONCERN: LOW /HIGH/UNCLEAR**

Low

DOMAIN 2: INDEX TEST(S)

If more than one index test was used, please complete for each test.

A. Risk of Bias

Describe the index test and how it was conducted and interpreted:

2. PCR: Total DNA extraction was performed with commercial DNA extraction according to the manufacturer's protocol. DNA was used to perform conventional or quantitative PCR with primers specific to mycobacteria. For conventional PCR the intensity of the band was used to identify the PCR product with molecular weight relative to positive control. The quantitative PCR (qPCR) result was considered positive to detect mycobacteria DNA until 40.0 cycle threshold (Ct).

- ❖ Were the index test results interpreted without knowledge of the results of the reference standard? Yes — Yes/No/Unclear
- ❖ If a threshold was used, was it pre-specified? Yes — Yes/No/Unclear

Could the conduct or interpretation of the index test have introduced bias? **RISK: LOW /HIGH/UNCLEAR**

Low

B. Concerns regarding applicability

Is there concern that the index test, its conduct, or interpretation differ from the review question? **CONCERN: LOW /HIGH/UNCLEAR**

Low

DOMAIN 3: REFERENCE STANDARD

A. Risk of Bias

Describe the reference standard and how it was conducted and interpreted:

According to the Brazilian Ministry of Health guidelines, the SSS was executed and is taken from 4 routine sites of dermal scraping samples from earlobes and at least one elbow and/or typical skin lesion. Bacterial index (BI) counting and morphological analysis were used in a common optical microscope.

- ❖ Is the reference standard likely to correctly classify the target condition? Yes — Yes/No/Unclear
- ❖ Were the reference standard results interpreted without knowledge of the results of the index test? Yes — Yes/No/Unclear

Could the reference standard, its conduct, or its interpretation have introduced bias? **RISK: LOW /HIGH/UNCLEAR**

Low

B. Concerns regarding applicability

Is there concern that the target condition as defined by the reference standard does not match the review question? Low **CONCERN: LOW /HIGH/UNCLEAR**

DOMAIN 4: FLOW AND TIMING

A. Risk of Bias

Describe any patients who did not receive the index test(s) and/or reference standard or who were excluded from the 2x2 table (refer to flow diagram):

Not applicable.

Describe the time interval and any interventions between index test(s) and reference standard:

The standard reference test and the index tests were collected, processed and analyzed at the same time.

- Was there an appropriate interval between index test(s) and reference standard? Yes — Yes/No/Unclear
- Did all patients receive a reference standard? Yes — Yes/No/Unclear
- Did patients receive the same reference standard? Yes — Yes/No/Unclear
- Were all patients included in the analysis? Yes — Yes/No/Unclear

Could the patient flow have introduced bias? **RISK: LOW /HIGH/UNCLEAR**

Low
